# Speech Recognition in Natural Background Noise

**DOI:** 10.1371/journal.pone.0079279

**Published:** 2013-11-19

**Authors:** Julien Meyer, Laure Dentel, Fanny Meunier

**Affiliations:** 1 Linguistics Division, Museu Paraense Emilio Goeldi (MPEG), Belém, Brazil; 2 Universidade Federal do Para (UFPA), Belém, Brazil; 3 Laboratoire sur le Langage, le Cerveau et la Cognition (L2C2), CNRS, UMR5304, Institut des Sciences Cognitives, Lyon, France; University of Salamanca- Institute for Neuroscience of Castille and Leon and Medical School, Spain

## Abstract

In the real world, human speech recognition nearly always involves listening in background noise. The impact of such noise on speech signals and on intelligibility performance increases with the separation of the listener from the speaker. The present behavioral experiment provides an overview of the effects of such acoustic disturbances on speech perception in conditions approaching ecologically valid contexts. We analysed the intelligibility loss in spoken word lists with increasing listener-to-speaker distance in a typical low-level natural background noise. The noise was combined with the simple spherical amplitude attenuation due to distance, basically changing the signal-to-noise ratio (SNR). Therefore, our study draws attention to some of the most basic environmental constraints that have pervaded spoken communication throughout human history. We evaluated the ability of native French participants to recognize French monosyllabic words (spoken at 65.3 dB(A), reference at 1 meter) at distances between 11 to 33 meters, which corresponded to the SNRs most revealing of the progressive effect of the selected natural noise (−8.8 dB to −18.4 dB). Our results showed that in such conditions, identity of vowels is mostly preserved, with the striking peculiarity of the absence of confusion in vowels. The results also confirmed the functional role of consonants during lexical identification. The extensive analysis of recognition scores, confusion patterns and associated acoustic cues revealed that sonorant, sibilant and burst properties were the most important parameters influencing phoneme recognition. . Altogether these analyses allowed us to extract a resistance scale from consonant recognition scores. We also identified specific perceptual consonant confusion groups depending of the place in the words (onset vs. coda). Finally our data suggested that listeners may access some acoustic cues of the CV transition, opening interesting perspectives for future studies.

## Introduction

Speech-in-noise research has revealed that speech signals incorporate several acoustic properties that contribute to compensating for signal distortions and noisy interferences. For example, they include enhanced spectral peaks for vowels, rapid spectral changes for consonants, amplitude modulation patterns to highlight informative portions such as stress or vowel-consonant alternations, or periodicity of the waveform perceived from any harmonic of the signal (see Assman and Summerfield [1] for a review). In parallel, our cognitive system was found to be adapted to overcome speech degradations and is able, to some extent, to overcome distortions and fragmentations of the signal. This means that our perceptual and cognitive systems perform highly sophisticated mechanisms of informational shielding [2], [3]. As a result, speech recognition remains possible even after large amounts of the signal have been removed, such as via gating in the time domain (e.g., [4]), drastically filtering the frequency domain (e.g., [5], [6]), or significantly altering the spectro-temporal coherence [7]. Even when the spectral details and periodicity of voiced speech are eliminated, intelligibility remains high if the temporal modulation structure is preserved in a small number of frequency bands [8].

**Figure 1:**
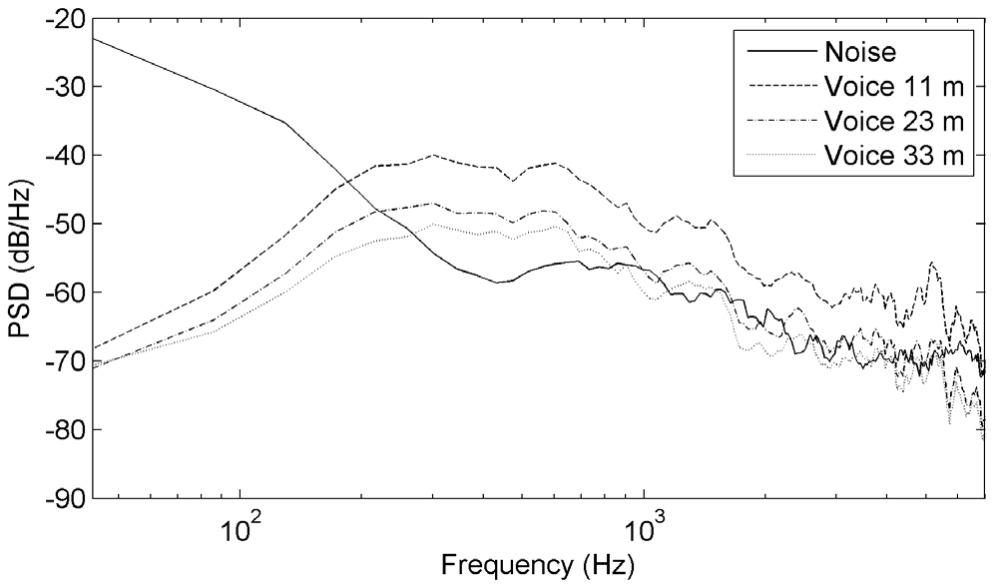
Long-term spectrum of the interfering noise and of spoken voice at three distances of the experiment. Power spectral density (PSD) as a function of frequency.

The emergence and the evolution of these adaptive listening abilities in humans have developed in rural environments which are the dominant setting for the vast majority of human evolution. Hence, the acoustic constraints present in these environments are interesting to take into account in order to study human speech recognition in conditions approaching ecologically valid contexts. So far, there is no systematic study dealing with the impact of natural acoustic backgrounds on spoken recognition. One difficulty explaining this situation is that rural background noise is known to be rather variable even when it does not include mechanical sources of noise. It depends on the geographical situation, the terrain, the vegetation, meteorological circumstances, but also bio-noises such as animal calls and hydro-noise such as rivers or sea rumble. However, natural background noises have common underlying basic properties that are different from the ones used in most speech-in-noise experiments (such as periodic tones, random noises, artificial broadband noises, artificial continuous and fluctuating noises, or speech-shaped noises [1]). One reasonable solution would therefore be to focus primarily on the most regular and frequent acoustic constraints encountered outdoors. Such basic constraints are characterized by a non uniform distribution of noisy frequencies. A first important characteristic is that it emphasizes low frequency content and therefore resembles the frequency distribution of pink noise at this level. A second important aspect to note is that the power levels decrease more rapidly than the ones of pink noise as a function of increasing frequencies. Moreover, at higher frequencies the power level distribution rather resembles speech-shaped noises (see Figure 1 and ‘materials and methods’).

Until now, the studies concerning the influence of outdoor natural environments on speech have focused on three main domains: first, on the human ability to tacitly adjust vocal output to compensate for intensity losses due to sound propagation over distance, known as the Lombard effect [9], [10]; secondly, on the human ability to estimate the distance of the speaker [11], [12]; and finally, on the natural adaptation of the speech signal into shouted speech forms or into other alternative acoustic media, such as whistled forms of languages that enable dialogue over long distances [13]. Interestingly, distance has been an essential ecological parameter implied in all these studies. Namely, this parameter is suitable to reveal the impact of ambient noise which progressively merges with speech during the spoken signal transmission. Listening to distant speech is a rather common task in daily life, both in urban or rural contexts. However, the great majority of speech-in-noise recognition studies available in the literature thus far have concerned close listening conditions. There are few papers testing speech recognition with distance and they are mostly targeted at testing indoor environments such as classrooms [14], halls [15], or even tunnels [16]. Speech recognition with distance has recently sparked new scientific interest in the fast developing domain of indoor human-machine environments, where a whole set of sound capture techniques and algorithms of signal treatment have been developed for automatic speech recognition [17]. In such cases, the speech signal is not only affected by the ambient noise but is also degraded during its in-air transmission between the speaker and the listener.

In the present study, we took a complementary approach to former experiments, by evaluating the ability of normal-hearing individuals to recognize words and their constituent phonemes at variable distances in a very basic model of an outdoor environment. For a first study of this type we decided to test relatively stationary and long-term natural acoustic effects of such environments and, as noted above, we decided to explore their most frequent and regular acoustic constraints. The speech signal was masked by a natural background noise recorded in a flat open field characterized by very low - assumed to be negligible - reverberation indices. Therefore, distance was simulated in the simplest way by amplitude attenuation only. This method had the advantage of corresponding to variations in signal-to-noise ratio as in most speech-in-noise studies existing in the literature. For each participant several listening distances were tested, as lists of target isolated French words were played between the virtual distances of 11 m and 33 m from the participants. Word recognition performance was the first measured parameter. An intelligibility function was derived from these results. Next, we analysed recognition performance on vowels, consonants and syllable structure, underlining the differences between different classes of phonemes and ranking them as a function of recognition performance. Our results extend some findings of other speech perception studies to the specific conditions of the present study. This was the case for the central role played by vowels in word detection as well as for the strong relationship between consonant recognition and the identification of the lexical meaning of isolated words. We also found peculiarities in natural background noise, particularly concerning confusion patterns of phonemes. We explained them by combining perceptual and acoustic analyses as a function of parameters such as distance ( = SNR levels), position of the consonant in the word, or phonetic features such as place, manner or voicing.

## Materials and Methods

### Participants

The 36 participants were 18 to 30 year-old French native speakers. Their normal hearing thresholds were tested by audiogram. The present study was conducted in accordance with the Declaration of Helsinki. It was approved by the ethics committee of the SPIN research group (CNRS) and each participant gave written consent.

### Stimuli

In total, 19 lists were recorded in a sound-proof box by a masculine speaker trained for this task in the DDL-CNRS laboratory (mean level of words was 65.3 dB(A) at one meter from the speaker, with a standard deviation of 3.3 dB). Each list contained 17 French isolated words. The French language is characterized by a certain balance between vowels and consonants that contributes to avoiding drastic numerical asymmetry that might favor computations of consonants over vowels [18]. The selected words were nouns regularly used in current French vocabulary. They were mostly monosyllabic words, and a few - less than 5% - were words of CVV and VVC syllabic structure. For all lists, all participants and all simulated distances, the distribution of the played word structures was as follows: 82.1% for CVC, 12.7% for CCV, 4.1% for CVV, 0.8% for VVC, and 0.3% for VCC.

Moreover, all the lists were balanced in terms of:

Frequency of word occurrence in the French language: the average word frequency per list was between 3.79 and 3.91 according to the evaluation method of New et al. [19].
Number of phonological neighbors for each word. This number was on average between 19.59 and 20.1 for each list.
Number of phonemes per word. The average for each list was between 4.5 and 4.6 letters.
Duration of pronunciation of each word. The average duration of the words in each list was between 547 to 553 ms.
Alternation between vowels and consonants. Each list contained in average the same number of possible CVC alternations.
Gender of the nouns. There was approximately the same number of masculine and feminine nouns in each list.

Each list was organized on a single audio track where each word was separated from the following by 3 seconds of silence. All these tracks were calibrated with the same root mean square energy level. From these original audio tracks we built new audio files by applying the masking effect of the background noise and the amplitude attenuation simulating distance.

## Design and Procedure

### Background noise

The natural background noise interfering with the speech signal was recorded in a flat open field (near Vilanova i la Geltru, Spain). The recording precautions enabled us to capture a relatively stationary background noise (standard deviation of 1.2 dB) in low level conditions (mean value of 41.6 dB(A), measured with a sound level meter BK 2240). This ambient noise was chosen because it was representative of diurnal background noises typically found in rural isolated geographic areas, with quiet weather and no noisy animal near the recorder. Acoustically, such noises are characterized by high energy levels at lower frequencies of the voice spectrum (below 300 Hz) and a strong decline towards higher frequencies (see Figure 1). Their frequency-dependent distribution of acoustic energy levels is explained by the fact that absorption increases with frequency in natural environments due to the terrain, the vegetation, and the micro-climates that noisy signals traverse [20].

#### Distance simulation

In a natural environment, the impact of background noise on speech recognition is revealed by distance. As mentioned in the introduction, we chose the simplest method to simulate distance: by amplitude attenuation, applying the inverse square law for outdoor spherical propagation. Word lists were presented at different levels corresponding to the attenuation simulation for each distance and they were masked by the selected background noise (Table 1) in accordance with the reference levels measured during the recording sessions of the speaker and of the noise. Therefore the variable distances resulted in variations of the SNR according to distance. It was a relatively realistic option as background noise recordings were made in quasi-stationary meteorological conditions (wind speed <1 m/s throughout the session, degree of humidity between 57% and 65%, temperature between 26°C and 28°C, measured on a portable meteorological station Geos Skywatch), one meter above from the ground, in an open field made of a plain dirt track which is the guarantee of low reverberation indices.

**Table 1:** Absolute amplitude levels and SNRs of words played at each distance of listening.

Distance (meters)	11	13	15	17	19	21	23	25	27	29	31	33
Level (dB(A))	44.5	43	41.8	40.7	39.7	38.9	38	37.3	36.7	36	35.5	34.9
SNR (dB)	−8.8	−10.3	−11.5	−12.6	−13.6	−14.4	−15.2	−15.9	−16.6	−17.2	−17.8	−18.4

With a reference mean value of 65.3 dB(A) for words at 1 meter from the source and a natural background noise produced at 41.6 dB(A).

#### Signal-to-Noise Ratio

The SNR levels were estimated by calculating the sound power levels of all lists played at each distance (we concatenated words without silent pauses between them and applied the Welch's method [21], [22]) and by subtracting from these values the sound power levels of the long-term frequency spectrum of the selected noise (cf. Table 1). The calibration of the listening equipment in the laboratory was made with a 2 kHz reference sinewave recorded on the field in the natural background noise and for which we measured sound power level in dB(A) at 1 meter from the source. We also checked the values of two reference words recorded and measured on the field in the same conditions.

#### Procedure of the experiments

Each participant sat in front of a computer in the experimental studio of the Institut des Sciences de l'Homme of Lyon (CNRS, University of Lyon) and was asked to perform the test played on specialized software interface and delivered diotically via headphones (Beyerdynamic DT 48, 200 X, with free field equalizer, according to [23]). All computers were identical with identical sound cards and had been calibrated for the experiment according to the reference measures of the original recordings. The participants had the simple task of listening to each stimulus and trying to recognize the isolated target word, in an open response format. They were asked to type the perceived sounds, even if they did not correspond to a French word, into the software interface through the computer keyboard and then validate their answer in order to move on to the following word (the experiment can be tested through the interface given in Supporting Information, see Protocol S1). The participants did not receive any feedback on their performance before the end of the test. After a training phase of 5 words to ensure that they had understood the task, the test phase began with a list of 17 words. For each participant, a different list was presented at each of the 12 distances tested. We either increased the distance progressively from 11 meters to 33 meters (with a two-meter step), or decreased it progressively from 33 meters to 11 meters. The reason why we chose these two presentation options instead of randomizing distance was that we wanted to verify whether there was a differential impact on performance between a progressive distance increase and a progressive distance decrease. However, such a distinction was not found (F(1, 11) = .94; n.s.).

## Results

First, we will present the general results of word intelligibility. Then, we will provide a detailed analysis of recognition performance for various phonological properties, such as syllabic structure, phoneme type and consonant position in the word. This large set of data will enable us to detect some specificities of the effect of natural background noise on speech recognition. Most of our results are based on recognition percentage scores. In order to stabilize variance and normalize proportional data, an arcsine transformation [24] was applied to such scores before running inferential analyses.

### General word recognition performance

**Figure 2:**
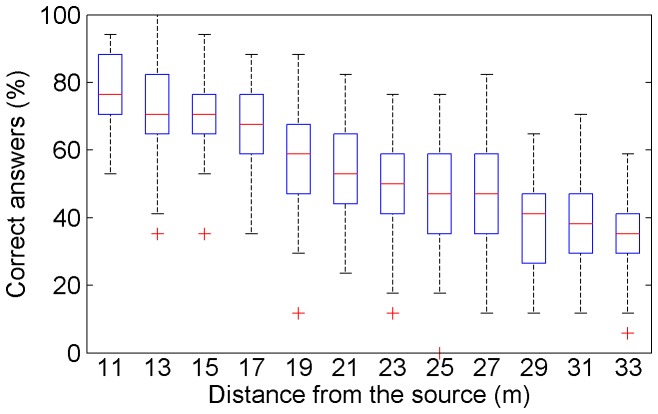
Word recognition as a function of distance.

The mean word recognition performance was 54.6% of correct answers for all the participants at all distances. The performances showed a general decrease in the average proportion of correct answers, from 77.8% at 11 meters to 35.9% at 33 meters, with consistent inter-individual variability at each distance (Figure 2). There was a strong quasi-linear correlation between distance and the intelligibility loss (R^2^ = .95). The SNR categories associated with each distance enabled us to derive the values of the speech reception threshold (SRT) and the corresponding slope of the empirical intelligibility function (across lists and participants). The SRT value was found at −15.2 dB of SNR, and the slope at 3.2%/dB (values calculated on more than 600 words in each category of SNR levels that straddled the 50% correct answer point). The intelligibility values also showed that the experiment mostly concerned the central quasi-linear part of the psychometric function, which corresponded to the SNRs most revealing of the progressive effect of the selected noise.

### Word structure recognition, phoneme insertion or deletion

**Figure 3:**
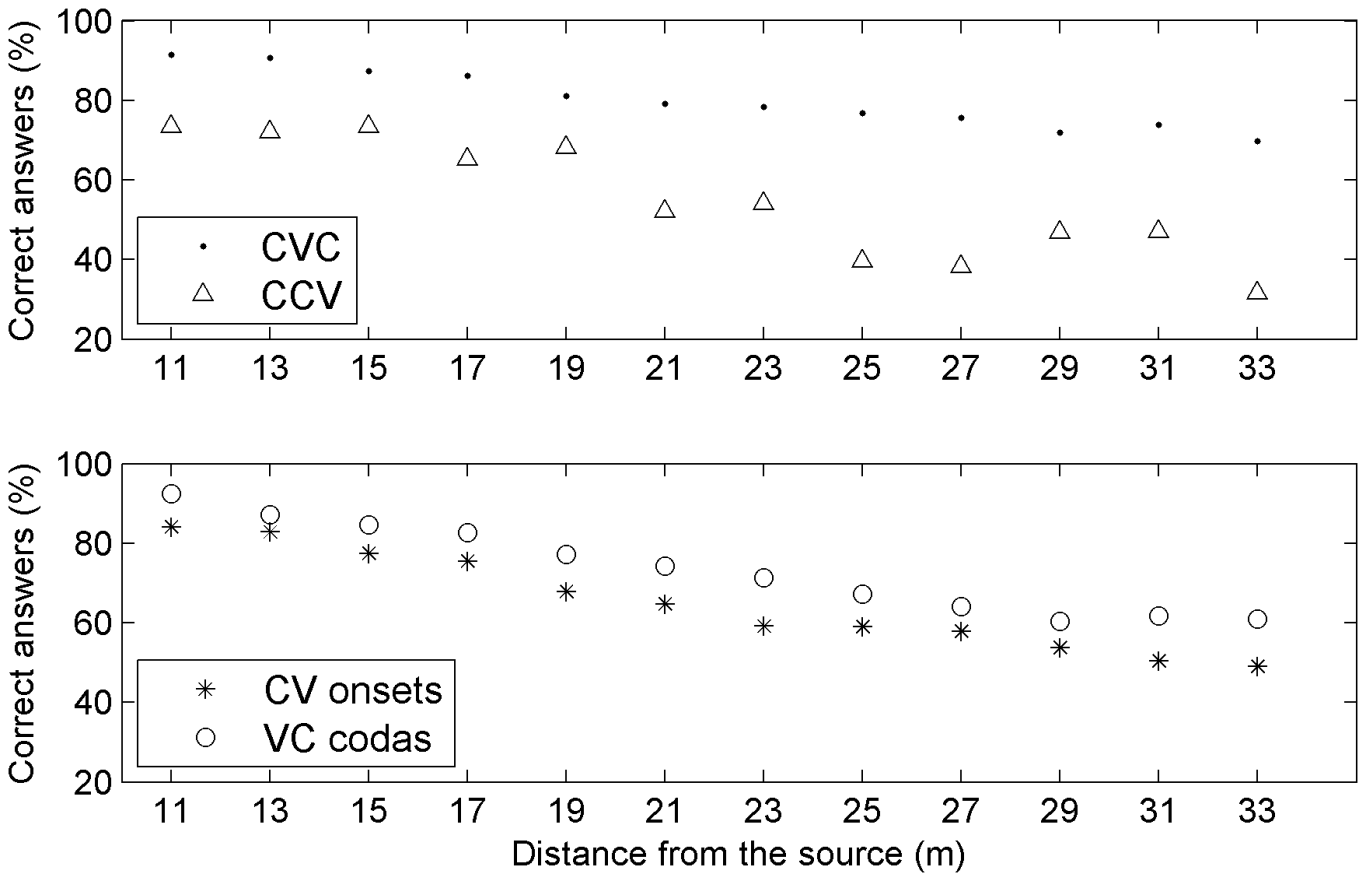
Distance effect for CVC and CCV syllabic structures (high), as well as for CV onsets and VC codas in CVC (low). CVC were much more frequent and therefore the evolution with distance is more regular than for CCV data.

The mean recognition performance for the syllabic word structures of the corpus was 76.5% of correct answers, over all distances. The two most frequent structures were CVC, which was recognized at 80.2%, and CCV, which was recognized at 55.7%. The progressive degradation in recognition performance with regards to distance confirms CVC as a very resistant syllabic structure (Figure 3). In general, structural errors were of two main types: phoneme deletion(s) or phoneme insertion(s). Deletions and insertions could occur at the same time. Deletions were much more frequent than insertions. Some deletions were due to an absence of response for the entire word (20.6% for all structures, 23.6% for CVC words, and 12.9% for CCV words). When looking only at the errors which were not due to an absence of response for the entire word, deletions remained prevalent. Indeed, in this case, 73.6% of all non recognized structures were errors involving deletions (73.3% for CVC and 78.2% for CCV), whereas the errors with insertions reached 36.8% (34.7% for CVC and 37.9% for CCV). The proportion of words involving deletions increased monotonically with distance from 11 m (8.7%) to 33 m (27.5%), except between 19 and 21 meters where a greater step occurred. Moreover, the proportion of words involving insertions increased quasi-linearly from 4% at 11 m to 12.9% at 33 m. Both deletions and insertions were more frequent with consonants than with vowels: consonants were involved in 85.5% of the insertions and in 98.7% of the deletions (involving respectively 7.4% and 17.3% of all words). Another aspect we checked was the impact of the position of the consonant in the word. The most interesting case was for deletions in CVC words, which occurred a little more on onsets than on codas (9% vs. 7% of all words). In fact, codas were more accurately perceived at any distance (see the lower section of Figure 3).

### Vowel recognition and confusion

**Figure 4:**
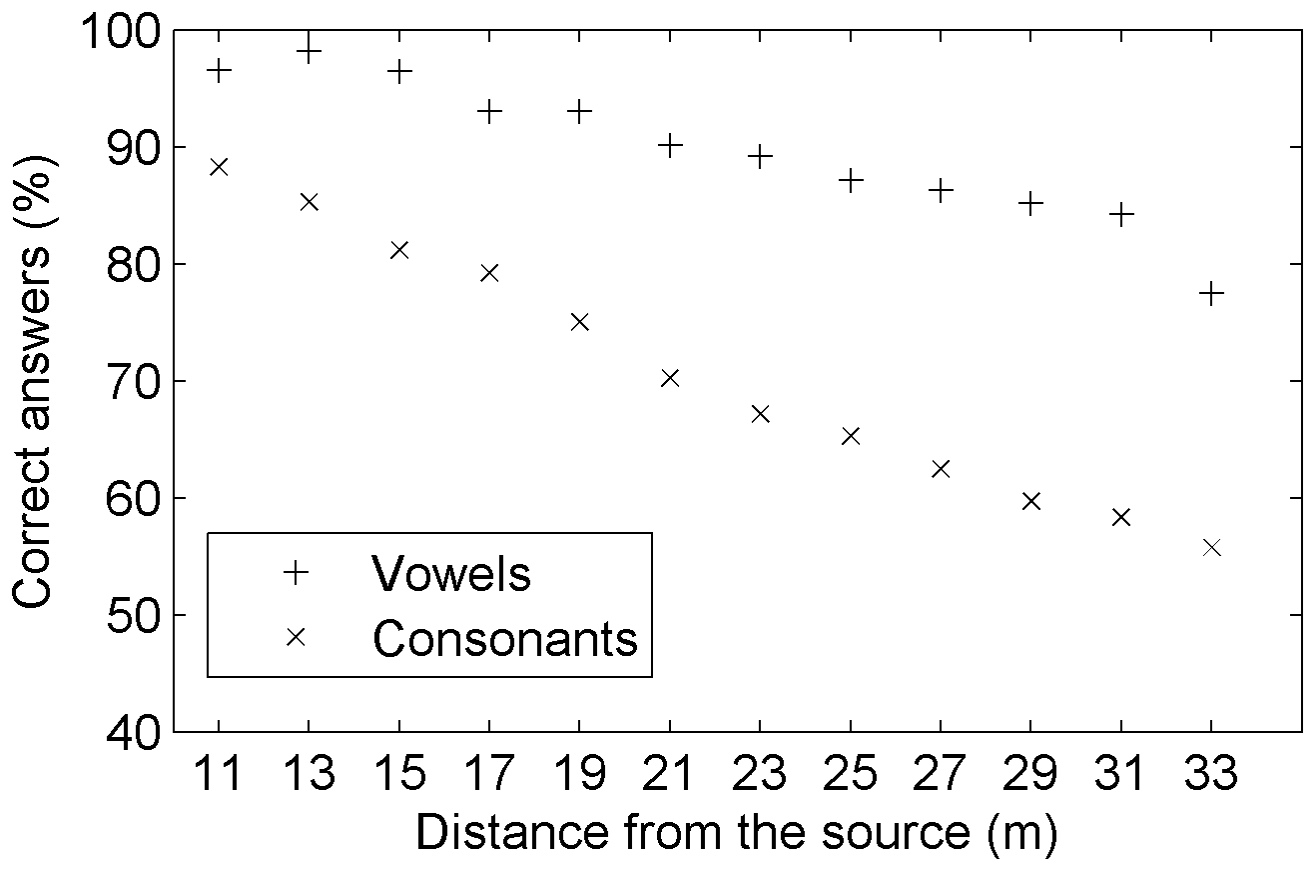
Distribution of errors (deletions vs. confusions) for vowels (left) and consonants (right). Data is presented either as a function of individual phonemes (up) or as a function of distance (bottom).

**Figure 5:**
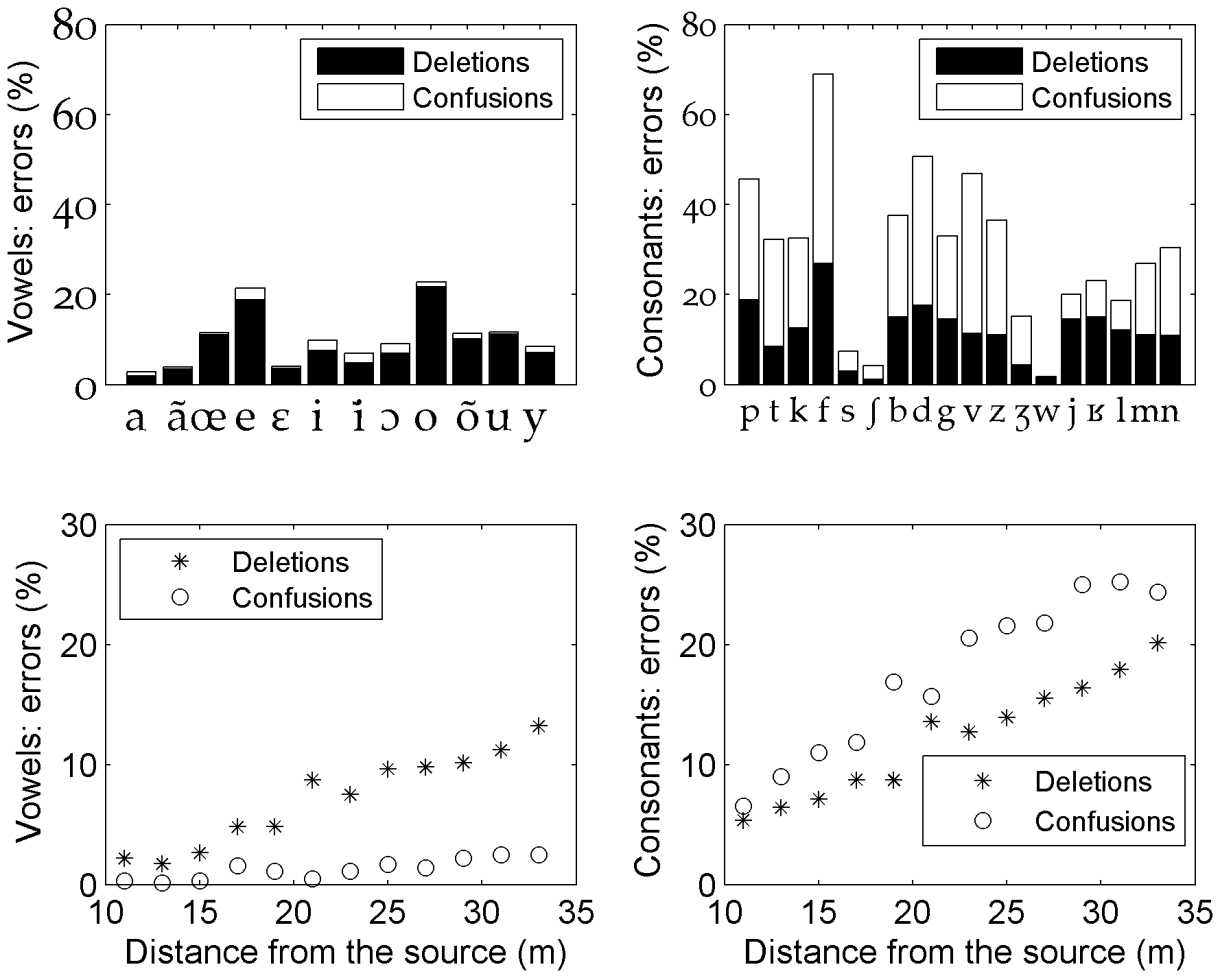
Recognition performance on vowels and consonants. The correct answers are presented as a function of distance.

The mean vowel recognition performance - taking into account all vowels at all distances - was very high (91.5%). Among the 8.5% of errors, a great majority was due to an absence of a response (deletions) (85%) and much fewer were due to confusions with other vowels (15%) (Figure 4, top left). All vowels pooled together, the recognition scores as a function of distance remained above 90% up to 23 meters and above 80% up to 33 meters (Figure 5). Vowel deletions increased more rapidly than vowel confusions as distance increased. A greater variability on recognition scores also appeared at greater distances. Strikingly, deletions of vowels corresponded nearly always to an absence of response for the entire word (98.6% of the cases). The position of the vowel in the word didn't change much the results (for CCV words the mean vowel recognition performance was 90%). Moreover, we found some variability between vowels. For example, the vowels [a, 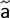, 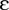] were best recognized (with over 96% of correct answers), whereas [e, o, 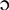] had the lowest recognition performances (with 79%, 77% and 72.3% of correct answers, respectively). Some vowels were also more often confused than others, however, among the different types of vowel confusion, none were significantly more frequent than others.

### Consonant recognition

#### General results

The mean recognition performance for consonants was 70.3%, across all distances and all consonants. We observed a decrease in the average proportion of correct answers from 88.1% at 11 meters to 55.5% at 33 meters (Figure 5). We also observed a strong variability between consonants (Figure 4, top right). The post alveolar approximant [w] appeared only as [wa] or [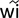] preceded by another consonant (CCV contexts), which explains its very high level of recognition (98.2%). For this reason, we excluded it from further analyses. However, we kept other consonants that were frequent in the second position of CCV contexts, such as [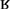, l], because their recognition scores in the CVC contexts were similar to those in CCV contexts. Among the 29.7% of errors across all consonants and all distances, 58.8% were due to confusions with other consonants and 41.2% were due to the absence of a response (deletions). The general predominance of confusions over deletions remained valid at all distances (Figure 4, bottom right). However, for liquids and approximants, errors were more often due to deletions than to confusions (Figure 4).

### Consonant recognition as a function of distance and SNR

**Figure 6:**
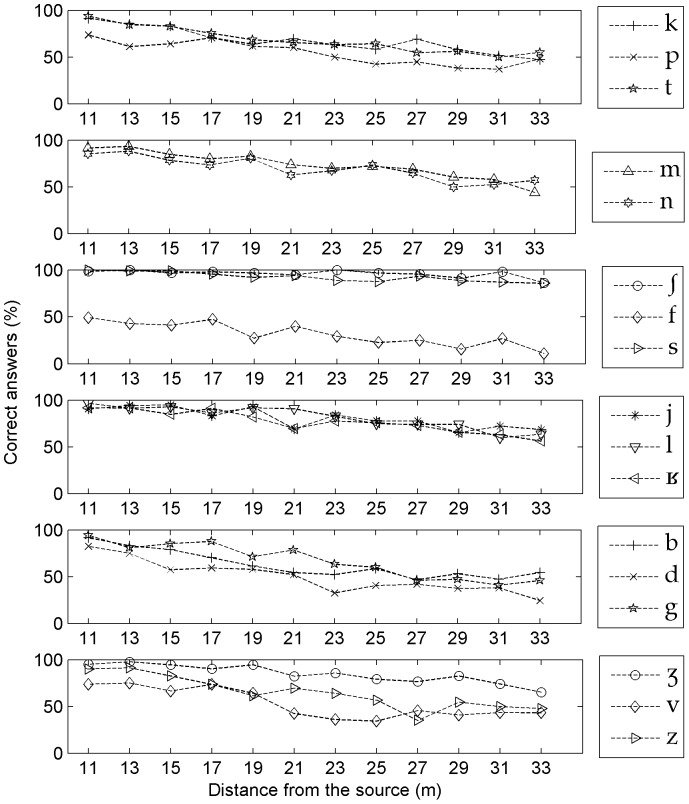
Recognition of the 17 most played consonants as a function of distance.

Looking more specifically at how distance affects recognition performance of the 17 most common consonants [p, t, k, f, s, 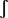, b, d, g, v, z, 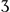, 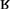, l, j, m, n], we measured the average correct answers associated with each of them at each of the 12 tested distances (Figure 6). We also established a tentative ranking of consonants, from the best to the worst recognized (1), based on simple criteria such as mean general recognition scores and distance-dependent drops in recognition performance. We also used the distance-dependent 50% threshold of recognition as an additional criterion to distinguish consonants that had the same ranking along the former two criteria (see Figure 6 for details). Finally, we obtained the following ranking:
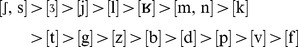
(1)This ranking underlines two important frequency acoustic characteristics influencing recognition scores: sibilant frication and formant like resonance patterns (also called sonority). The sibilant frication of [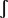, s, 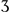 has the advantage of remaining above the natural background noise, of being narrow band and also of falling within the range of the best perceived frequencies in human hearing [25]. But frication is not the whole story: for example the sibilant [z] ranked in the low middle part of the scale (1). The parameter distinguishing [z] from [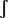, s, 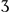] is the relatively intense amplitude of the frication of the latter [26]. Therefore, the quality of sibilant frication was found to be an important factor in recognition performance, but it must be associated with a high energy level to provide a good transmission performance, such as for [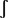, s, 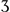]. At the same time, energy alone cannot explain the scale (1), simply because [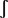] and [s] were better recognized than all vowels and semi-vowels which are by far the most energetic phonemes. To write scale (1) in a different manner we identified the common features characterizing the consonants in question, as follows:
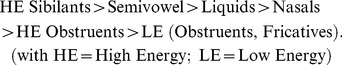
(2)


### Consonant confusion

With the intention of clarifying consonant confusion patterns, we analysed them in detail by taking into account three principal factors: consonant position, phonetic features they carry and distance corresponding to SNR variations.

#### Confusion matrix

**Figure 7:**
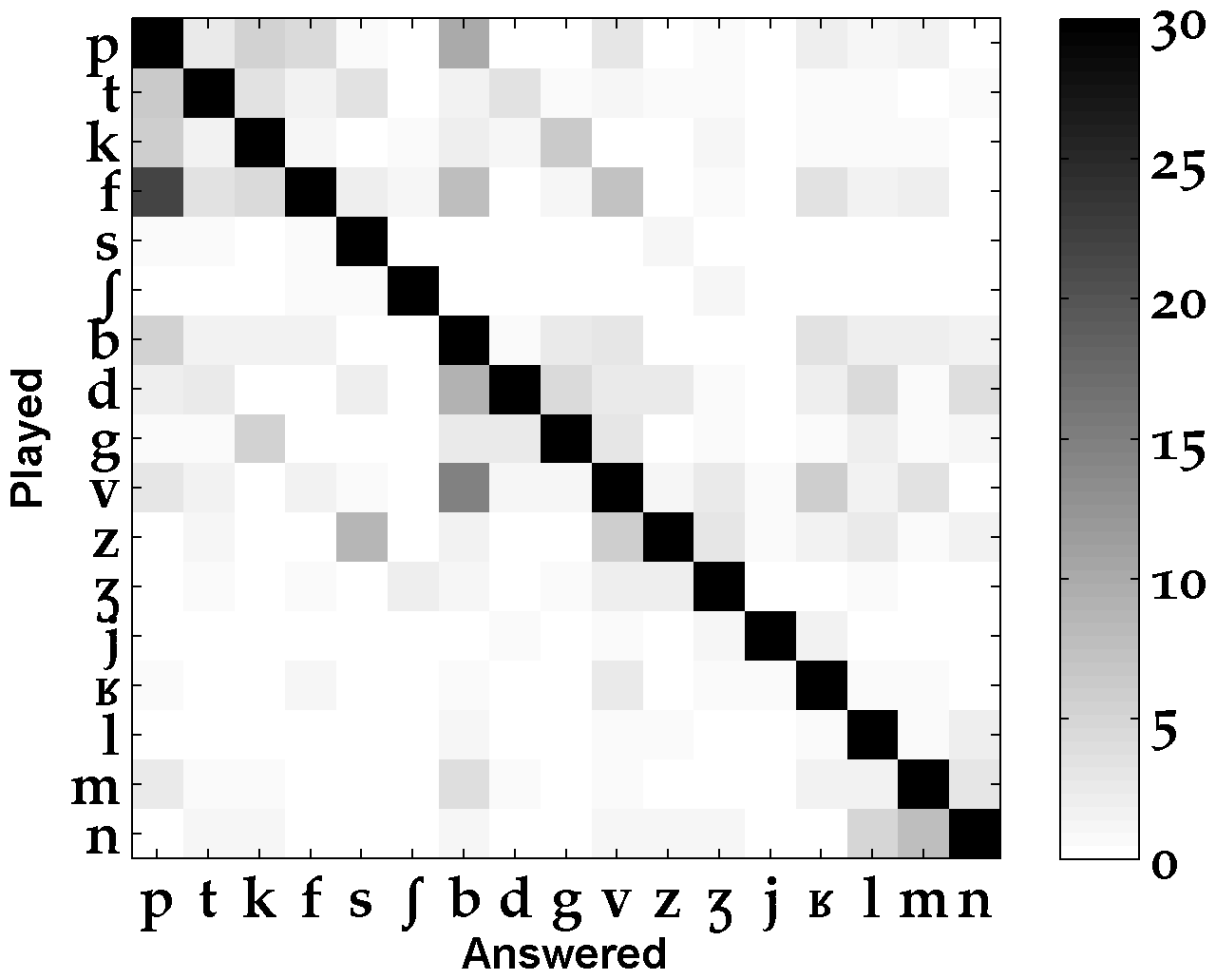
Confusion matrix for 17 of the French consonants (% of confusions).

In a first step, we measured the instances of confusion between the 17 consonants [p, t, k, f, s, 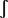, b, d, g, v, z, 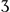, 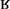, l, j, m, n]. A confugram (Figure 7) shows an overview of these results, with all distances pooled together. These general measures revealed that the confusions mostly involved consonants sharing at least one phonetic feature related to place or to manner of articulation. For example, the three most frequent inter-consonant confusions concerned the situations when: [f] was taken for a [p], [p] was taken for a [b], [k] was taken for a [g].

### Influence of consonant position

**Figure 8:**
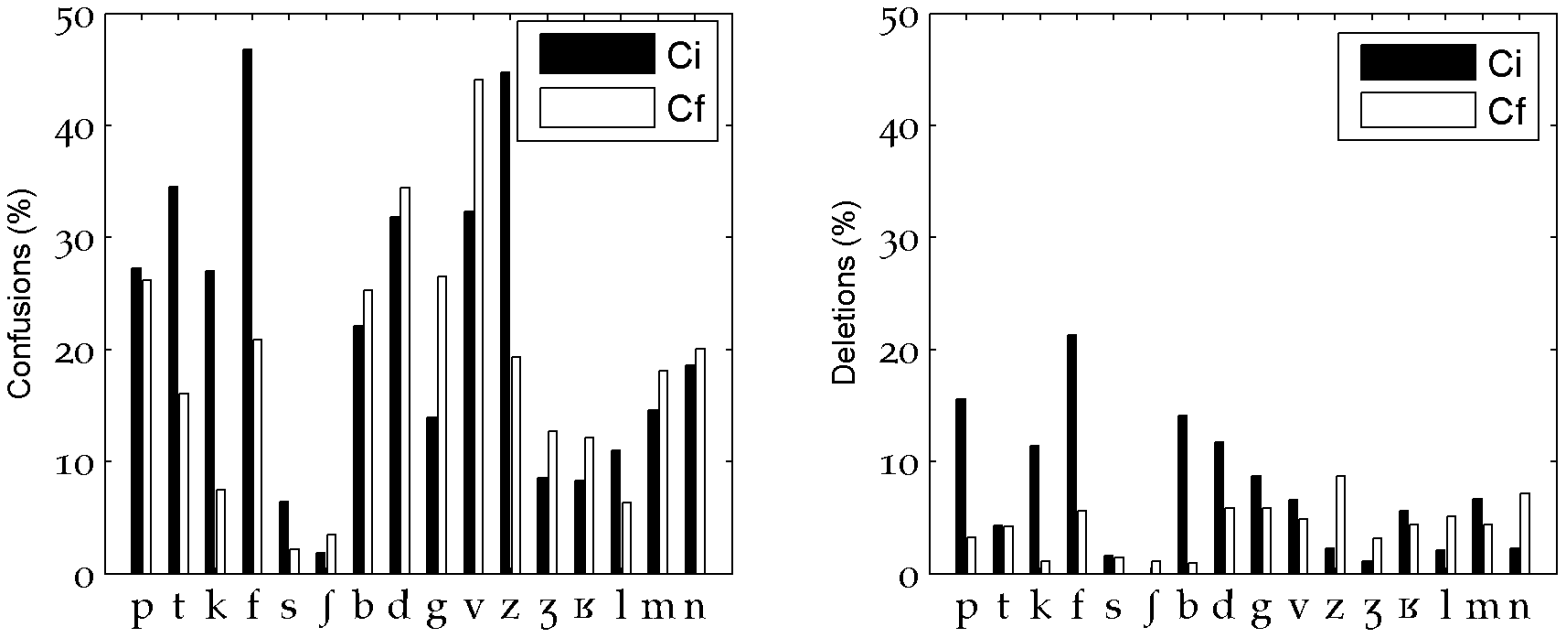
Proportion of confusions (left) and deletions (right) in onsets (Ci) and codas (Cf). Displayed values show the % of played consonant for each type.

A more detailed observation of the instances of confusion revealed that some consonants were more likely to be confused when they were at the beginning of the word (such as [t, k, f, s, z]), whereas others were more confused when they were at the end of the word (such as [b, d, g, v]) (Figure 8). One of the consonants, the approximant [j] appeared only at the end of words and was therefore excluded from some of our further analyzes separating onsets and codas. Moreover, the position of the consonant in the word often influenced the nature of the confusion: [t] was mistaken for a [d] only in onsets, whereas [d] was mistaken for a [t] only in codas. A similar phenomenon occurred in the [k]-[g] pair where the unvoiced [k] was mistaken for the voiced [g] only in onsets and [g] was mistaken for a [k] mostly in codas. This unvoiced-voiced relationship was not systematic as the [p]-[b] confusions almost always occurred in onsets (92.6%), showing that the labial locus of articulation is less subject to asymmetry in recognition. In fact, of the three voiced stops, [b] is known to be the most compatible with sustained voicing [27], [28].

**Figure 9:**
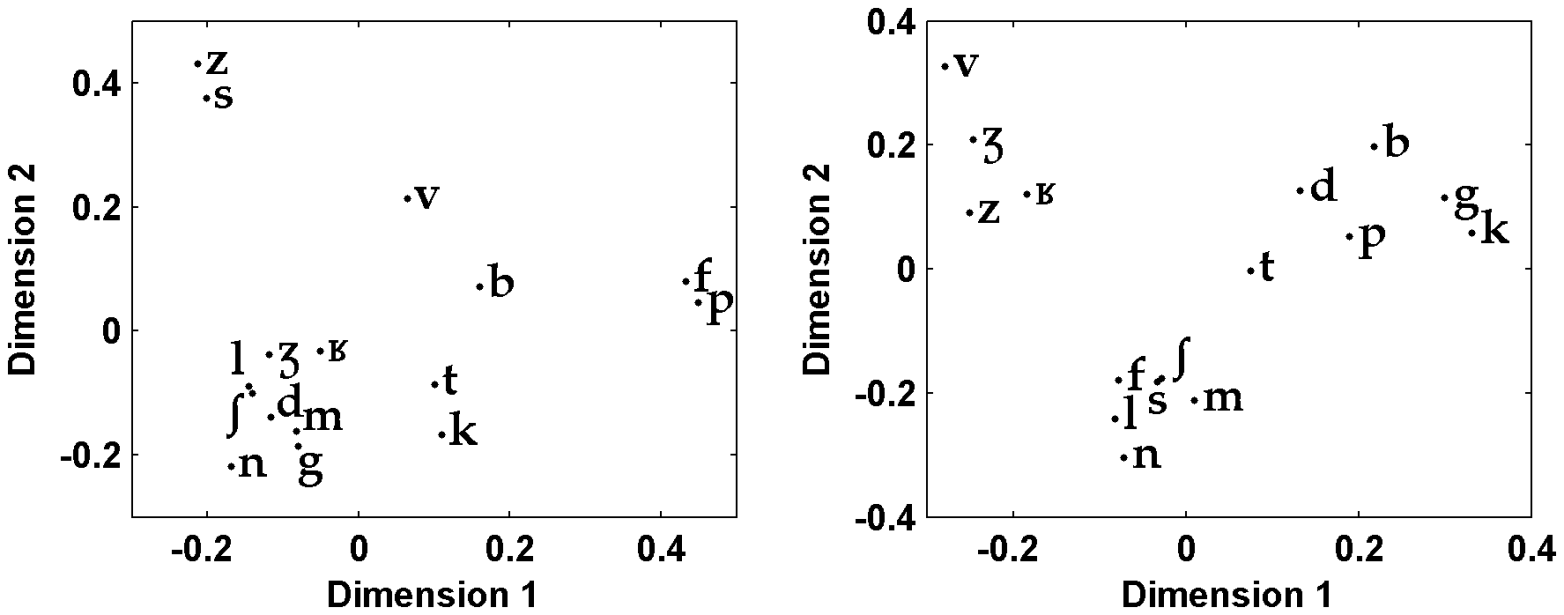
Perceptual mapping of consonant confusions in onsets (left map) and codas (right map).

In order to develop a general perspective on the consonant confusions in onsets and codas, which represented together 99.4% of the confusions in our corpus, we used multidimensional scaling and mapped the perceptual space that caused the confusions in these positions (called also Ci - for initial - and Cf - for final -). We applied Shepard's [29] method of psychological representation of speech sounds to calculate consonant similarity based on confusion matrices and mapped their corresponding perceptual distances (Figure 9, left and right). One of the first things we can see in these maps is that they illustrate different general patterns for Ci and for Cf. The map relating to Ci is not very clear (Figure 9, left), yet some logic in the disposition of the phoneme emerged as a large area in the upper right quarter of the map contains the labial place of articulation [v, b, f, p]. Other groupings are due to the influence of intense confusions in particular pairs of phonemes: for example [n] and [m], [k] mistaken for a [t] or a [g], [s] mistaken for a [z], and [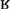] often confused with labials. Moreover, on this Ci map, the voiced consonants are nearly always situated to the left of their unvoiced counterpart, emphasising the fact that voiced forms are better recognized. Finally, the fricative form is found higher up, and it is on the left when better recognized. On the other hand, the perceptual mapping in Cf (Figure 9, right) shows three very clear regions associated with similarities in responses to certain groups of consonants: one on the top right containing plosives, a second one on the top left containing voiced fricatives and the trill, and a third one on the bottom containing unvoiced fricatives, nasals and approximants. An interesting observation is that, while mapping these similarity judgments, we tested various configurations and found that the perceptual groupings didn't change much when restricted to some distances only, for example at distances above 23 meters where recognition scores are low.

### Manner, place and voicing

Guided by the preceding results, we computed the information associated with some of the constituent phonetic features of consonants, using information transmission as the recognition metric, as defined by Miller and Nicely [30]. In order to compute the amount of information transmitted, the sixteen consonants of the recognition set were partitioned into three (overlapping) groups on the basis of voicing, articulatory manner and place of articulation (as illustrated in Table 2). We derived confusion matrices for each phonetic-feature dimension from the original confusion matrix by summing the results for each feature group. In essence, each phonetic-feature dimension was treated as an independent information channel.

**Table 2:** Consonant groups on the basis of the phonetic properties of voicing, manner and place of articulation.

Features	Values	Members
Manner	Fricative	[f, s, <$>\raster(70%)="rg2"<$>, v, z, <$>\raster(70%)="rg3"<$>]
	Nasal	[m, n]
	Plosive	[p, t, k, b, d, g]
	Approx.	[<$>\raster(70%)="rg4"<$>, l]
Place	Labial	[p, b, f, v, m]
	Coronal	[t, d, s, <$>\raster(70%)="rg2"<$>, z, <$>\raster(70%)="rg3"<$>, n, l]
	Dorsal	[k, g, <$>\raster(70%)="rg4"<$>]
Voicing	Voiced	[b, d, g, v, z, <$>\raster(70%)="rg3"<$>, <$>\raster(70%)="rg4"<$>, l, m, n]
	Unvoiced	[p, t, k, f, s, <$>\raster(70%)="rg2"<$>]

**Figure 10:**
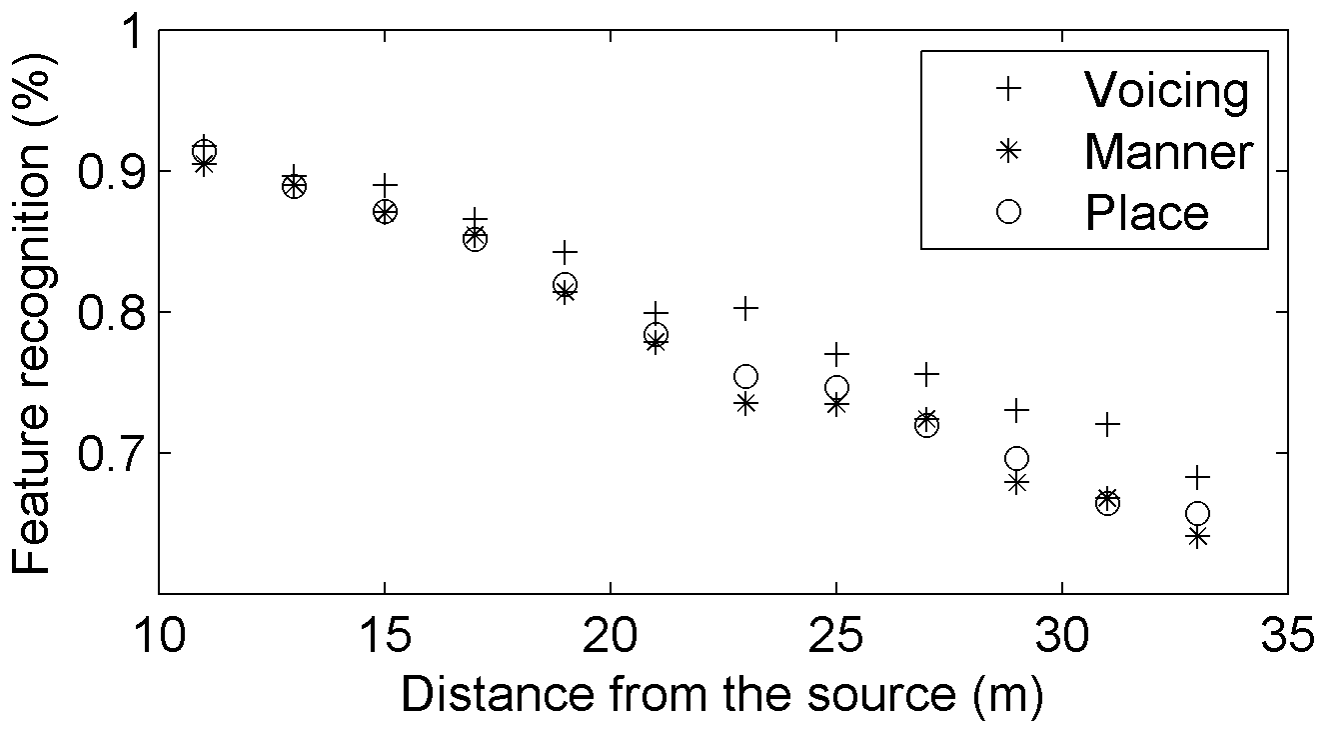
General recognition performance for the three main categories of features (voicing, place, manner).

For all distances pooled together, voicing was the best recognized with 80.6% of correct answers, followed by place (78.1%) and manner (77.4%). We also measured the recognition performance for the feature categories and subcategories as a function of distance. First, we found that manner, place and voicing were recognized at equivalent levels until 21 meters (−14.4 dB of SNR). Above 21 meters, voicing was better recognized than place and manner (manner being slightly less well recognized, see Figure 10). Additional measures showed that this was the case both in Ci and Cf, with the strongest effect in Cf.

**Figure 11:**
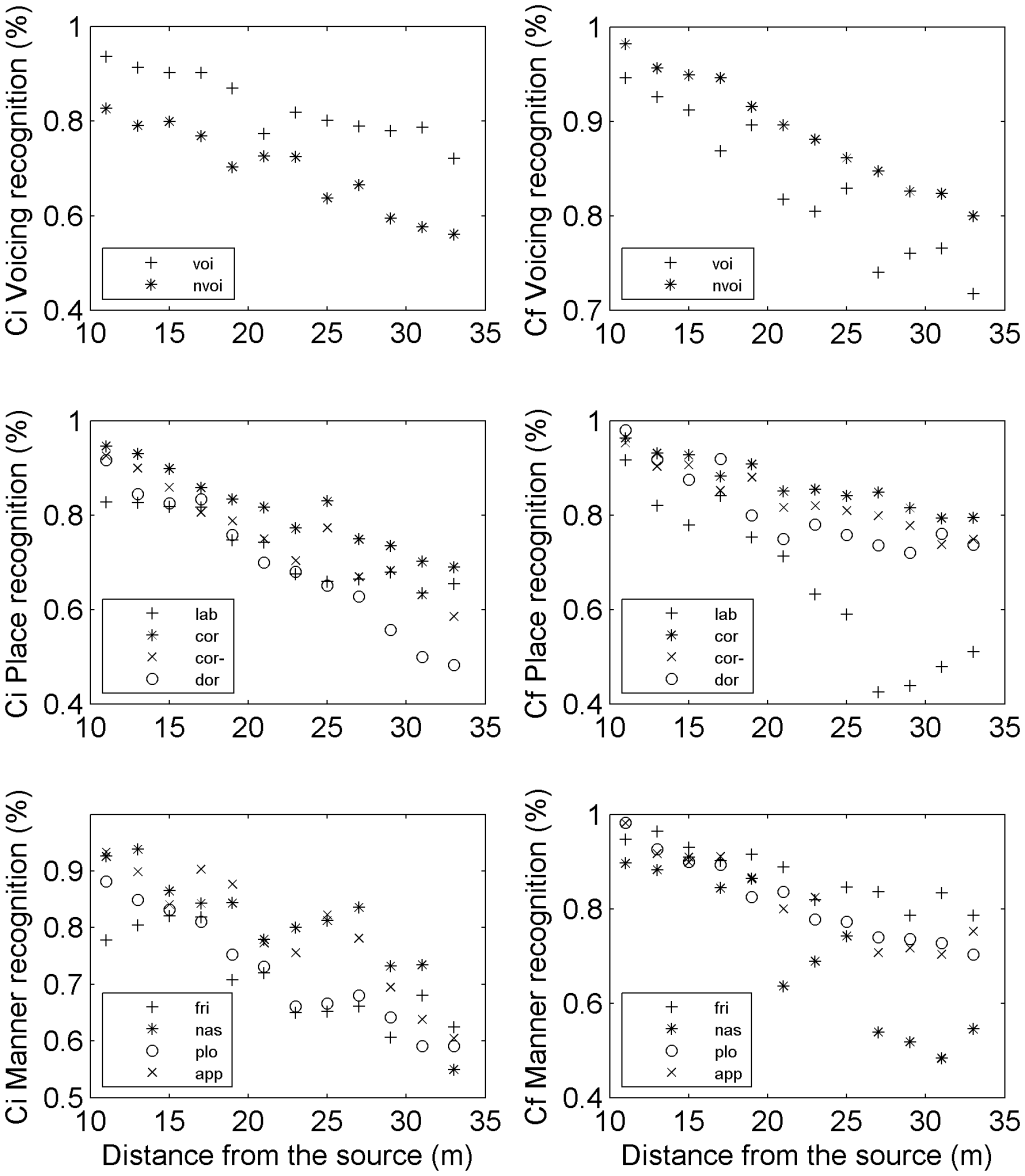
Recognition of voicing (up), place (middle), and manner (bottom), in Ci (left) and Cf (right). The labels are: ‘voi’ for voiced, ‘nvoi’ for unvoiced, ‘lab’ for labials, ‘cor’ for coronals, ‘cor-’ for coronals without [s, 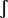], ‘dor’ for dorsals, ‘fri’ for fricatives, ‘nas’ for nasals, ‘plo’ for plosives, and ‘app’ for approximants.

Next, we measured the recognition performance for each feature subcategory, with a distinction between Ci and Cf because of strong observed differences between these positions (Figure 11). In general, except for labials and nasals, recognition levels were better in codas than in onsets. Voiced consonants were clearly better recognized than unvoiced consonants at all distances in Ci (Figure 11, upper left part), whereas unvoiced consonants were systematically better recognized than voiced ones in Cf (Figure 11, upper right). Concerning place, coronals were the best recognized category both in Cf and Ci across most distances (cf. label ‘cor’ in Figure 11, center), even when the two powerful and highly recognized sibilants [s, 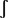] were excluded (cf. label ‘cor-’ in Figure 11). We observed a very striking phenomenon for labials in codas: their recognition performance dropped completely at distances over 21 meters (Figure 11, middle right part). This must be due to the fact that labial, coronal and velar contrasts are most perceptually salient in environments where the phoneme is released into a following vowel [31] and the labial acoustic cues in final positions do not fulfill salient cues like rapid frequency modulations or bursts. Concerning manner, nasals and approximants were generally better recognized in Ci than fricatives and plosives (except at 33 m, cf. Figure 11 bottom left part). In Cf, the fricatives were the best recognized overall (Figure 11, bottom right). A very striking phenomenon occured also for nasals in codas: their recognition performance dropped completely at distances over 21 meters, probably because of their slower spectral change relative to onset nasals (due to velum lowering during the vowel) [32], [33], [34].

### Phoneme Power Spectral Density (PSD) analysis

**Figure 12:**
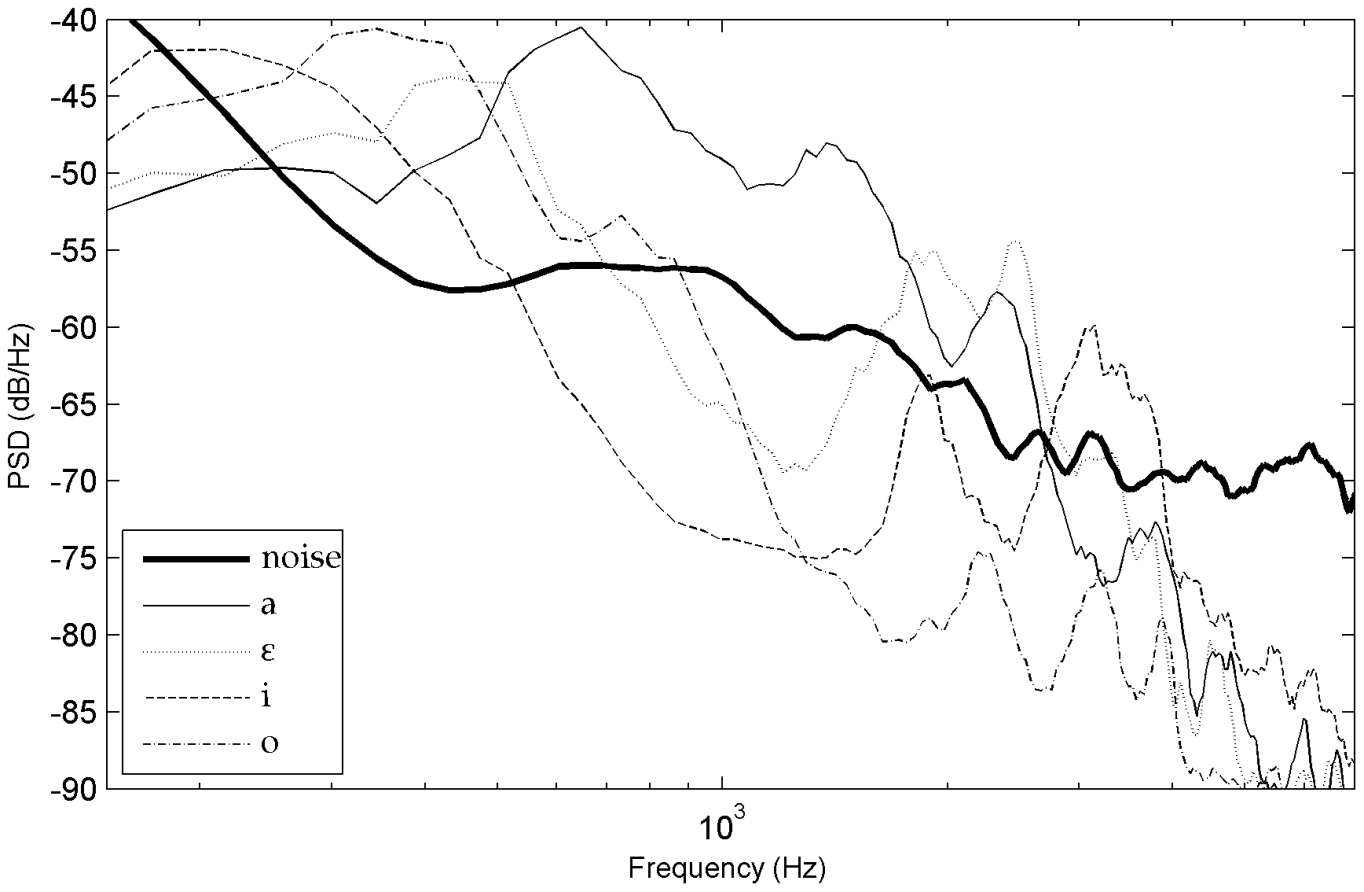
Power spectral density of the vowels [a, 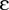, i, o] at 33 meters.

**Figure 13:**
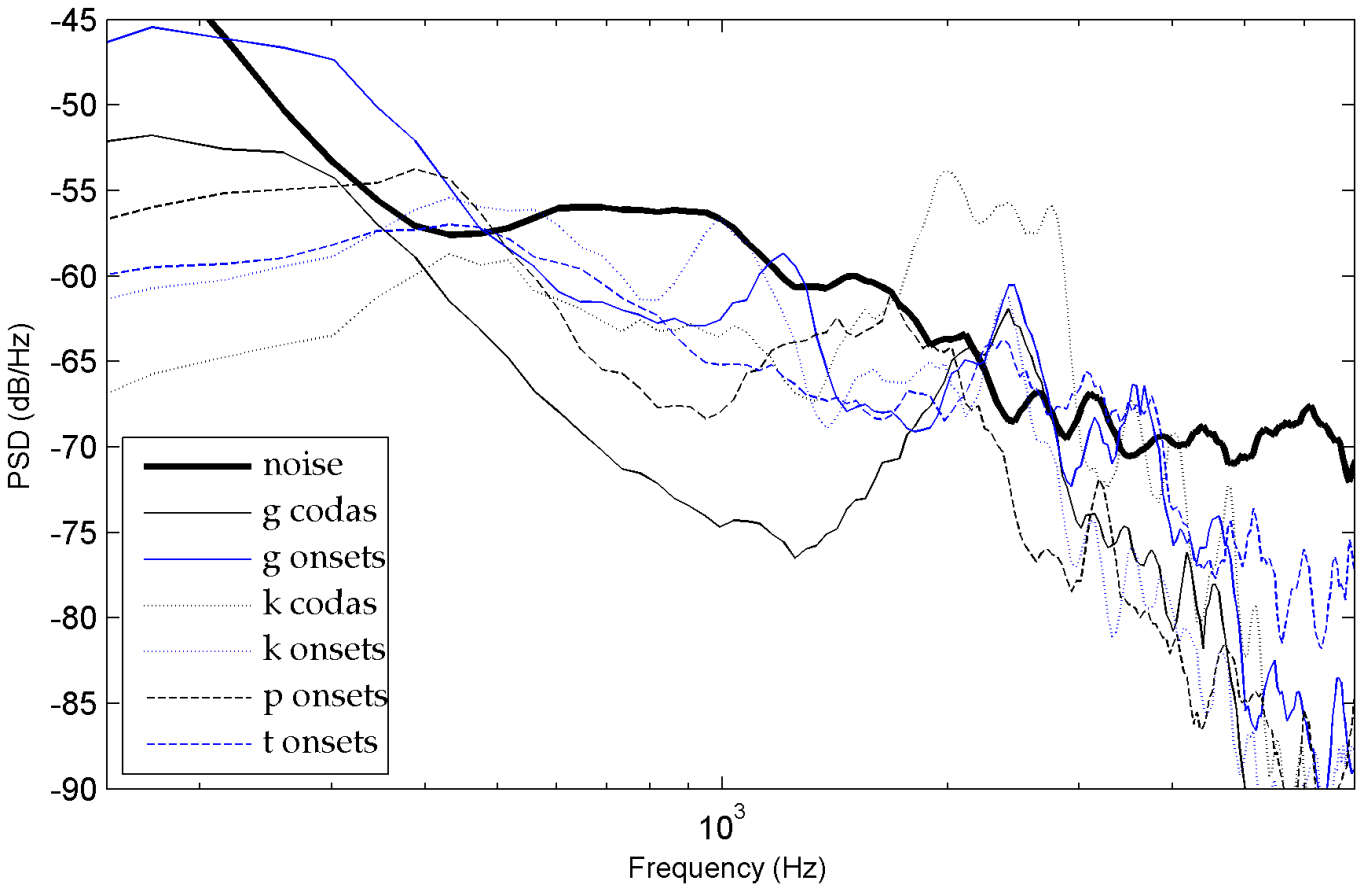
Power spectral density of some unvoiced plosives at 17 m.

Another important observation of this study was that phoneme recognition scores correlated in many respects with the SNR spectrum. In order to evaluate how the different acoustic characteristics of the phonemes interfered with the noise at variable distances, we contrasted their power spectral density with the power spectral density of the noise. The long-term power spectral density of phonemes was evaluated using sound extracts made of the concatenation of 10 items for each of the 17 consonant types (with a distinction between onsets and codas, except for [j] which was present only in codas) and of each vowel type [a, 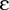, 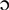, o, y, i, u]. We didn't evaluate the contribution of co-articulation as formant transitions were not included in the selected signals. In these conditions, it appeared that the acoustic cues associated with frication, formants, and burst sounds showed the most difference between phonemes and noise. At 11 meters, all vowels and consonants were characterized by strong phoneme-to-noise differences. On the contrary, at 33 meters, all vowels and only some consonants still had certain frequency bands above the noise. The results at 33 meters were particularly instructive for formants. Specifically, the 1^st^ and 2^nd^ formants of all vowels were highly prominent, except for [i] where the spectral peak of the 2^nd^ formant blended into noise (Figure 12). All vowels were also well recognized at this distance, even [i] which was recognized 101 times out of 121 (83.5%). In this particular case, the spectral peak formed by both the 3^rd^ and the 4^th^ formants of [i] was very prominent, confirming the important role of the F3–F4 close formants in the recognition of this front vowel [35]. For consonants, the salient frequencies at 33 m were mostly the frication of [s, 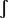, 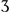], the bursts of plosives [k, g] and the 1^st^ formant in nasals [m, n] and in [l, j, g] (see some examples on Figure 13). These consonants were systematically associated with high recognition scores except for the ones which only had the 1^st^ formant above noise. Indeed, at 33 meters, the reduced recognition score of [m] (43%) contrasted with the moderately high recognition scores of [l, j] (63%, 68%) which had higher 2^nd^ or 3^rd^ formant patterns. This distinction suggests that listeners had access to other acoustic cues associated with formant peaks, particularly for [l] and [j] - perhaps formant transitions and/or rapid spectral changes - because the 2^nd^ or 3^rd^ formant peaks of [l, j, m] blended into noise at 33 meters.

**Figure 14:**
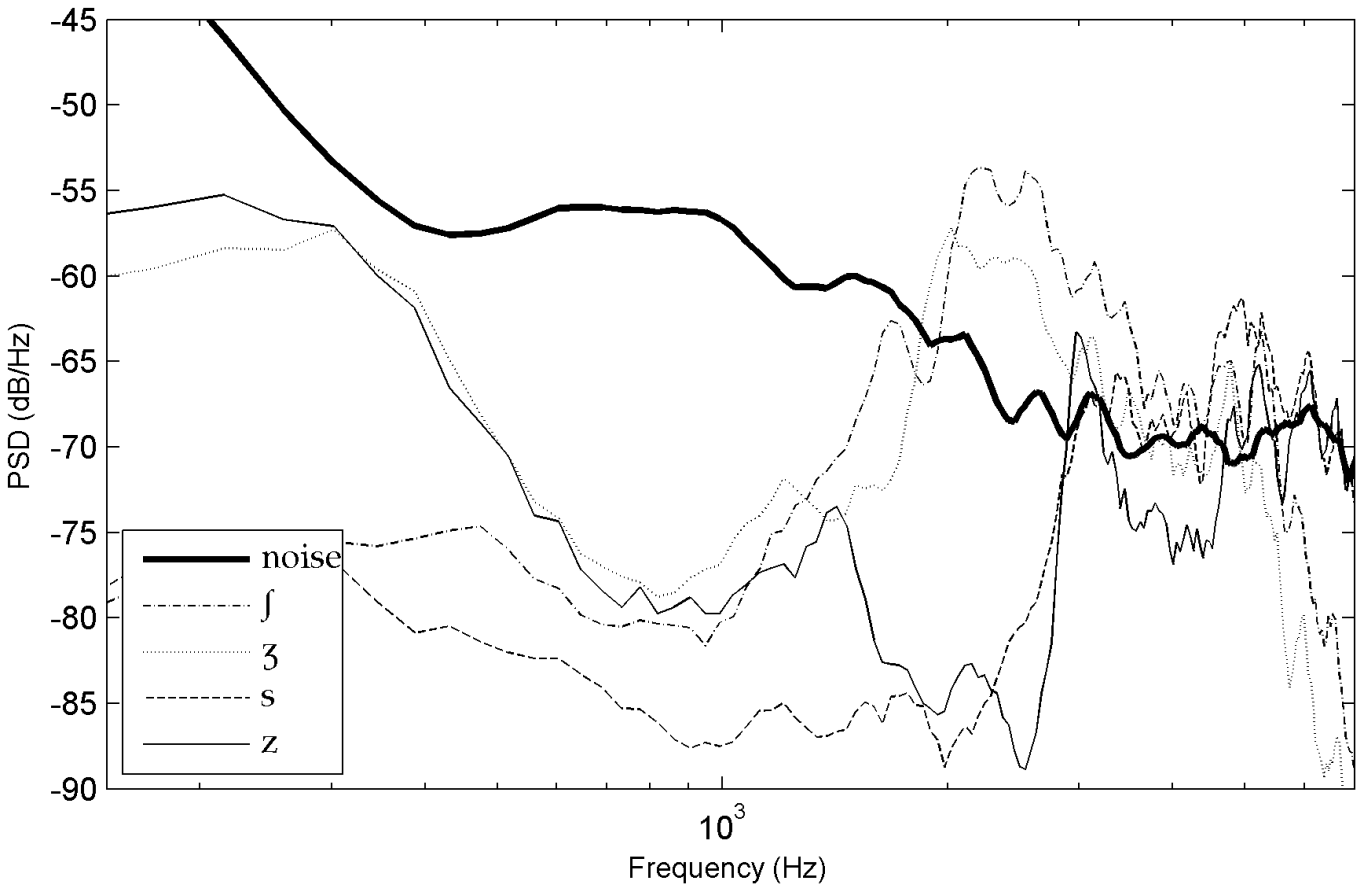
Power spectral density of several fricative consonants (onsets) at 33 m (codas are very similar).

In general, at every distance, the phonemes that had high recognition scores were the ones showing large differences with noise and therefore high amplitude levels of some of their characteristic acoustic cues. For plosives, we noted that the stronger the differences between the burst and the noise, the higher the recognition scores (for example, see the onset-coda differences of [g] and [k] in Figure 13). For fricatives, such a difference occurs for example between [s] and [z] (see Figure 14). Moreover, for stop consonants, when the bursts became unreliable because they were covered by noise, performances dropped around or below 50% (this was the case for [t] onsets at 27 to 33 m, whereas recognition scores for [t] codas remained above 65% because their bursts did not merge with the noise until 33 m). Other parameters that we didn't check systematically here may have also influenced the results for fricatives and plosives, such as probably the range of frequencies above noise and the rapidity of the CV spectral changes.

## Discussion

This study focused on the impact of slight natural background noise on speech recognition. We examined how such a noise interfered with word, phoneme and feature recognition as distance increased and we undertook an analysis of the cues that were used by the listeners for vowel and consonant recognition. Our simulation of distance was simple and resulted in changing the SNR (Table 1). The specificity of our study stems mostly in the non uniform frequency spectrum characterizing natural background noise and on the multiple ways in which we analysed its confrontation with the also non uniform distribution of energy in spoken voice frequencies.

### General word recognition

The intelligibility function classified the words as a function of distance and equivalent SNRs (Figure 2, Table 1). The SRT value derived from word recognition performance was quite low (−15.7 dB) because the noise didn't uniformly mask the speech signal. The slope corresponded to the average of other intelligibility tests with a paradigm based on isolated words (3.2%/dB) [36], [37]. The word recognition scores were higher than those found in the literature for non uniform pink noise at equivalent SNRs [38]. Intelligibility tests on sentences usually show steeper slopes (between 15 and 25%/dB according to [39]) indicating that the psychometric function is more precise. However, here, the advantage of choosing a paradigm with words in isolation was that it allowed for the assessment of confusion matrices and transmission of information: two aspects that we wanted to quantify, particularly for vowels and consonants.

### Syllabic structure effect

The recognition scores analysis on syllabic structure confirms CVC as a very resistant syllabic structure and revealed that errors on CCV and CVC were mostly due to deletions. Also, the perception of the final segments of words was improved and CV onsets were less well recognized than VC codas (Figure 3, bottom part): for example, more onset deletions occurred for 9 of the 16 consonants (Figure 8). This result is due to the lexical status effect that is characteristic of the choice of word stimuli [40], [41], whereas the tests based on artificial nonsense words (logatomes or pseudowords) systematically have better recognition performance on CV onsets (e.g., [40], [42]) and show better resistance of onsets to deletion.

### Differences between vowels and consonants

The recognition performances for vowels and consonants revealed various interesting differences between these two kinds of phonemes. First, vowels were, in general, far better recognized than consonants at any distance. This is in accordance with the literature on speech in noise perception involving white noise or speech-shaped noise ( e.g., [42], [43], [44]). The second striking difference was that errors on vowels and consonants showed opposite profiles: nearly all errors on vowels were due to an absence of response, whereas most errors on consonants were due to confusions, except for approximants (liquids and semi consonants) which had an intermediate behavior (Figure 4). All distances taken together, we found three times more errors and twelve times more confusions on consonants than on vowels. These proportions are unlikely to be specifically due to distributional properties because the CVC and CCV syllabic structures present only twice as many opportunities to produce similar sounding lexical neighbors due to consonants rather than vowels. Therefore, there was a higher stability of vowels over consonants in the kind of interfering noise we used, and this result strongly reinforces the idea that vowels and consonants play different roles in speech processing. Overall our results confirmed, first, the special functional role of consonants during lexical identification, but this time in speech-in-noise conditions. Next, it also confirmed that access to most of the vocalic acoustic cues providing information on the identity of vowels is preserved in such conditions. The literature concerning the respective roles of vowels and consonants in speech recognition is very prolific since the seminal study of Fletcher [45] on speech transmission. A recent perceptual experiment by Fogerty and Humes [46] dealt with CVC words, like in our experiment, and demonstrates that consonants contribute to intelligibility equally in both isolated words and sentences, whereas vowel contributions are mediated by context (with greater contributions to intelligibility in sentence contexts confirming that they may give key grammatical cues). Several aspects of our data gave indications about these different roles of vowels and consonants in speech processing. First, there was a very highly significant correlation (r = .99, p = 6.8e-11) between the progression of recognition scores for words and consonants as a function of SNR (respectively Figure 2 and Figure 5), whereas the correlation between the progression of recognition scores for words and vowels was lower (r = .94, p = 3.8e-6), but still high. Moreover, the mean and standard deviations of word recognition scores were closer for the ones of consonants than for the ones of vowels. These aspects argue for a strong relationship between consonant recognition and the identification of the lexical identity of words. This is in line with the idea that across a number of languages consonants constrain lexical selection more than vowels (see for example Cutler et al [47]). For vowels, an absence of response - the most frequent cause of error for this kind of phoneme- resulted in an absence of response for the word in 98.6% of cases. This occurred mostly when the masking effect of the noise was high, which means that the impression of having understood a word in an extremely noisy environment was conditional on the detection of the vocalic nucleus. This was even true when the constitutive consonants of the word were among the best recognized (such as [s] and [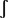]). It showed the central role played by vowels in the word-detection step that precedes the word recognition step in adverse conditions of listening. Indeed, as described in various previous studies, vowels have an energy advantage over consonants that make them robust and extraordinarily salient (e.g., [48]). Here, we observed that their formant pattern was only very progressively masked by a typical environmental background noise as distance increased, explaining their very high levels of recognition and low confusion rates compared to consonants.

### Acoustic cues and perception

The phoneme recognition and error data of this experiment was deployed as a function of different parameters such as distance, phonetic features or the position of the consonant in the word. Such data showed that formants, as well as sibilant and burst properties of phonemes were the principal parameters influencing performance of phoneme identification in natural background noise. The special roles of formant patterns and frication were first suggested by the consonant rankings (1) and (2) established on the basis of recognition performance. They were confirmed by the PSD analysis, which also highlighted the important role of bursts in stop consonants.

There are, in the literature, phoneme scales based on purely phonetic data that are in accordance with the perceptual ranking we found: for example scales ranking phonemes as a function of sonority (Clements [26]) but also scales ranking phonemes as a function of phonetic power [49]. Indeed, the presentation made in (2) strongly suggests that a scale based on perceived resonance (typically Semivowels >Liquids>Nasals>Obstruents, [26]) also influences the recognition scores in our study. Clements had explained that vowels stand at the top of such a sonority scale as they are characterized by a powerful, well-defined formant pattern and that sonorant consonants (that is, semivowels, liquids and nasals) are next on this scale as they are also characterized by a formant pattern but with a decreasing degree of definition. Moreover, the composite measure of the relative phonetic power of English sounds produced by Fletcher ([49], pp 82–86) fits with a sonority–based ranking, but also fits with the high rank of voiceless sibilants and with the low rank of low power fricatives found in (2).

**Figure 15:**
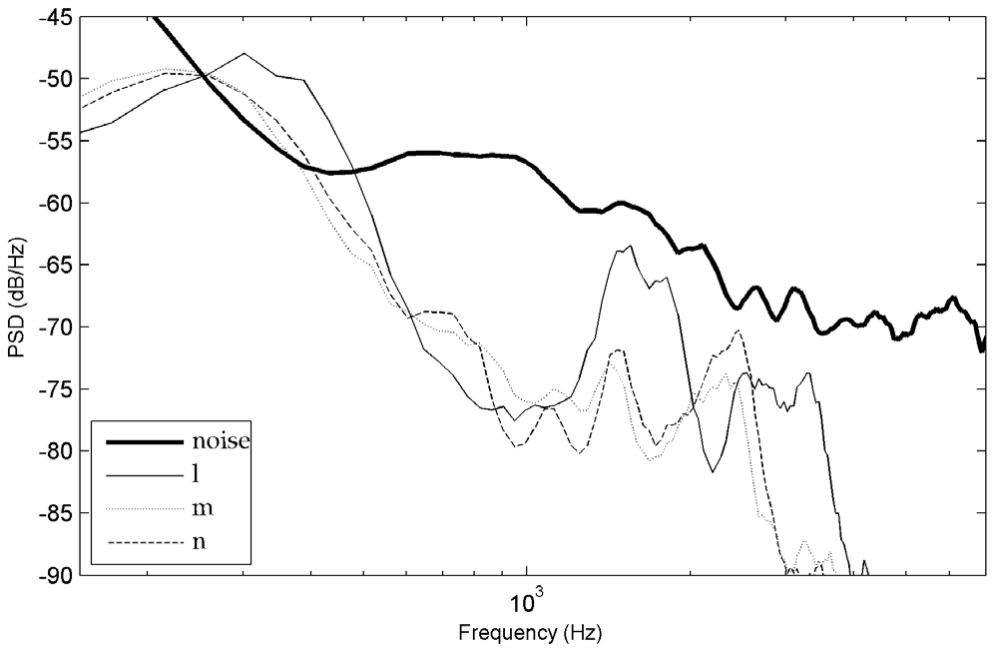
Power spectral density of [l, m, n] onsets at 33 meters (codas are very similar).

Our results showed that the most resistant consonants were [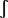, s, 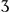]. This is in accordance with observations from speech recognition studies in speech-shaped noise, babble noise or white noise [50], [42], [43], [44]. For the rest of the consonants, the relative distribution of recognition levels found here resembled the ones of studies in white noise, but differed slightly from the ones obtained in speech-shaped noise and babble noise. For example, [t, z] were not high scoring as in Phatak and Allen [50] or as in Meyer and al [43] but rather intermediate scoring. Moreover, in these previous studies [f, v, b, m] were all low scoring, whereas here only [f, v] were low scoring but not [b, m] (except, [m] at 33 meters). Overall, the recognition levels we found were higher than the ones obtained at equivalent SNR levels in studies with broadband white noise or speech-shaped noise (e.g., [42], [43], [44]) and even with low-frequency noise [51]. One important reason for this difference is that the confusion rates on vowels, nasals and approximants were higher in these other types of noises because they masked much more the 1^st^ and/or 2^nd^ formants than our low level natural background noise. For example, confusions in babble-noise generally involve intense phonetic interference with acoustic cues of the concurrent speech signal, particularly formant patterns (e.g., [43], [52] which are studies based on the same word lists as here). Theoretically, low frequency components of the noise may mask higher frequencies of the speech signal [51], [53], but in the present study this would have concerned only the phonemes cues with the lowest sound differentials because the noise we chose is not strong (such as the 1^st^ formant of consonants [l, n, m] presented on Figure 15).

### Formants

The special role of formants underlined by vowel recognition and sonorant consonant recognition is in accordance with the descriptions that argue that such acoustic cues are well shielded against noise. For example, formants are known to be the main cues used by listeners for vowel recognition in noise [50], [54], [55]. Indeed, the narrow-band formant peaks in the envelope of the frequency spectrum generally provide robust cues for the recognition of sonorant phonemes, even when the spectral valleys are obscured by noise [1]. In the present study, the spectral peaks of the first vocalic formants were around 300 Hz or higher and therefore above the most energetic frequencies of the natural background noise. Moreover, the spectral peaks of the second vocalic formants included frequencies amongst the best perceived by human hearing and the PSDs we measured showed that the listeners still had access to clear information from this second vocalic formant for most of the vowel types (except for [i] at 33 meters which was recognized thanks to its F3–F4 peak, as noted in the result section). This was confirmed by the fact that no particular confusion occurred between vowels. On the contrary, in speech-in-noise studies using other kinds of noise, there are often vocalic confusions between vowels of close 1^st^ formant values but different 2^nd^ formant values because the 2^nd^ formant blends into the noise (e.g., [42], [56]). For consonants with formants (semivowels, liquids, nasals), the PSDs showed coherent formant patterns. However, these phonemes showed a reduced prominence of the second formant in comparison to vowels. This was in accordance with the literature [26], [57], [58]. For example, formants of liquids are characterized by a brief, intermittent, or narrow constriction in the oral tract that further reduces their spectral energy and prominence. We also found reduced formant peaks (F1 and F2) for [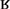] in comparison to [l] and also for [m] in comparison to [n]. These additional differences were coherent with differences observed in recognition scores.

### Consonant confusion groups

The clearest perceptual consonant confusion groups found in our study were ({b-v, f-p, f-b, m-b} {s-z} {p-k-t, k-g} {m-n}) in onsets and ({p-b, p-t-k, k-g} {m-n}) in codas. This was partly in accordance with the groups found by Benki [42] (which provided an onset/coda distinction for pseudowords in speech-dependent and flat-spectrum noise) or by Phatak and Allen [50] (which dealt with words in speech-shaped noise, without the onset/coda distinction that we made here). Similarities with Phatak and Allen [50] concerned mostly ({b-v},{s-z},{m-n}), whereas most of the preponderant confusions we found matched with those of Benki [42] except for the low level of confusion found by Benki between voiced and unvoiced plosives in codas. The perceptual consonant confusion groups that we found are characterized by an important occurrence of inter-labial confusions in onsets and of inter-stop confusions both in codas and onsets (these are for example clearly visible on the perceptual maps of Figure 9). The rapid merging with distance of the 2^nd^ formants of voiced consonants [b, g, m] partly explains these confusions. Moreover, for plosives, only bursts stood out of the noise past 19–21 meters. In these conditions, the close frequency values of [k, g, t] bursts must have contributed to their confusion.

## Conclusion

This paper shows how environmental noise and distance (from 11 to 33 meters) interfere with spoken word recognition. Our results showed that in such conditions, identity of vowels is mostly preserved and confirmed the special functional role of consonants during lexical identification. While vowels played central role in the word-detection step that precedes the word recognition step in adverse listening conditions, consonant seems mainly used to identify the lexical item. Also, we confirmed that CVC is a particularly resistant syllabic structure; we observed an asymmetry between the ending and the beginning of words, with CV onset being less resistant than VC coda. We also identified different perceptual consonant confusion groups depending of the place in the words: ({b-v, f-p, f-b, m-b} {s-z} {p-k-t, k-g} {m-n}) in onsets and ({p-b, p-t-k, k-g} {m-n}) in codas. Moreover, our data allowed us to propose a resistance scale were high energy sibilants are the more resistant, followed by semivowels and obstruents, and where low energy fricatives are the less resistant. These results clearly underlined the particular role of coherent strong energy structures like formants and sibilants in noisy situations. Complementary acoustic measures showed the important role of bursts in plosives. Finally, besides the acoustic cues of consonants and vowels that were identified as important for speech recognition, the study highlighted that listeners may have access to some acoustic cues of the CV transition that we didn't specifically track and that should be more completely investigated in the future in a complementary study using the same kind of noise.

## Supporting Information

Protocol S1 **General interface of the experiment.** The experimenter made the calibration with the calibration button on the left and then chose one of the two upper options on the right (increasing or decreasing distances). Next, each participant had the simple task of listening to each stimulus and trying to recognize the isolated target word (including in a preliminary training phase).
(RAR)
