# Correction: Speech Recognition in Natural Background Noise

**DOI:** 10.1371/annotation/012d9419-8135-40ab-8c81-ce46e8e708d0

**Published:** 2014-01-07

**Authors:** Julien Meyer, Laure Dentel, Fanny Meunier

This article was republished on January 6th, 2014 due to incorrect symbols appearing throughout the text. Please download the article again to view the correct symbols.

